# Cigarette smoke induces IL-8, but inhibits eotaxin and RANTES release from airway smooth muscle

**DOI:** 10.1186/1465-9921-6-74

**Published:** 2005-07-19

**Authors:** Ute Oltmanns, Kian F Chung, Matthew Walters, Matthias John, Jane A Mitchell

**Affiliations:** 1 Experimental studies National Heart & Lung Institute, Imperial College, London SW36LY, UK; 2 Cardiothoracic Pharmacology, National Heart & Lung Institute, Imperial College, London SW36LY, UK; 3 Department of Pneumology, University Hospital Charite, Berlin, Germany

## Abstract

**Background:**

Cigarette smoke is the leading risk factor for the development of chronic obstructive pulmonary disease (COPD) an inflammatory condition characterised by neutrophilic inflammation and release of proinflammatory mediators such as interleukin-8 (IL-8). Human airway smooth muscle cells (HASMC) are a source of proinflammatory cytokines and chemokines. We investigated whether cigarette smoke could directly induce the release of chemokines from HASMC.

**Methods:**

HASMC in primary culture were exposed to cigarette smoke extract (CSE) with or without TNFα. Chemokines were measured by enzyme-linked immunosorbent assay (ELISA) and gene expression by real time polymerase chain reaction (PCR). Data were analysed using one-way analysis of variance (ANOVA) followed by Bonferroni's t test

**Results:**

CSE (5, 10 and 15%) induced IL-8 release and expression without effect on eotaxin or RANTES release. At 20%, there was less IL-8 release. TNFα enhanced CSE-induced IL-8 release and expression. However, CSE (5–30%) inhibited TNFα-induced eotaxin and RANTES production. The effects of CSE on IL-8 release were inhibited by glutathione (GSH) and associated with the induction of the oxidant sensing protein, heme oxygenase-1.

**Conclusion:**

Cigarette smoke may directly cause the release of IL-8 from HASMC, an effect enhanced by TNF-α which is overexpressed in COPD. Inhibition of eotaxin and RANTES by cigarette smoke is consistent with the predominant neutrophilic but not eosinophilic inflammation found in COPD.

## Background

Chronic obstructive pulmonary disease (COPD) is a major public health problem that is currently ranking as the fourth leading cause of death in the world [1]. It is characterised by progressive and largely irreversible airflow limitation associated with symptoms such as cough, sputum production, and dyspnea. A chronic inflammatory response of the lung to noxious particles, most notably tobacco smoke, but also occupational dusts and air pollution, is currently considered as the underlying pathological mechanism leading to this clinical condition [1]. However, the link between inhalation of harmful substances, such as cigarette smoke, bronchial inflammation and the development of airflow limitation is not completely understood.

Currently, cessation of smoking is the only intervention that slows down disease progression in COPD [2]. Although only a minority of smokers develop symptoms of COPD, there is evidence that even in the lungs of asymptomatic smokers the numbers of inflammatory cells are increased [3,4]. COPD is associated with the release and overexpression of many pro-inflammatory cytokines and chemokines including TNF-a and IL-8 [5]. Several studies have shown that cigarette smoke is capable of activating lung macrophages as well as resident lung cells such as epithelial cells and fibroblasts to release various inflammatory mediators including TNFα and the neutrophil chemokine, IL-8 [6-8]. These mediators together with proteases produced by activated neutrophils and macrophages are capable of sustaining inflammation and damaging lung structures. Specifically, the accumulation of neutrophils in the lung has been associated with more severe disease [9]. The precise mechanisms leading to neutrophil influx into the lungs of smokers remain unknown, but this may involve the release of neutrophil-specific chemokines such as IL-8.

By contrast, the airways of patients with allergic asthma are chracterised by a different profile of activated leukocytes. Unlike COPD, where neutrophils predominate, in asthma the eosinophil is present in large numbers, likely to be the result of eosinophil chemoattractants such as eotaxin or RANTES [10].

Human airway smooth muscle cells (HASMC) represent an important structural component of the airway wall. In addition to their traditionally accepted role as contractile cells, HASMC produce neutrophil and eosinophil chemotactic factors such as IL-8, eotaxin and RANTES [11-13]. The production of chemokines by these cells is of particular relevance considering the anatomical localization with proximity to the vasculature. Many substances capable of activating the airway smooth muscle synthetic capacity have been identified, mainly cytokines such as IL-1β, TNFα and TGFβ [11,14,15]. However, the effects of cigarette smoke on chemokine production from HASMC are not known.

Therefore, in this study we have exposed HASMC to cigarette smoke and assessed effects on the induction and release of the chemokines IL-8, eotaxin and RANTES.

## Methods

### Materials

Tissue culture reagents were obtained from Sigma (Poole, UK). Cell culture plasticware was purchased from Falcon Labware (Becton Dickinson, Oxford, UK). Recombinant human TNFα and matched antibody pairs for IL-8, eotaxin and RANTES enzyme-linked immunosorbent assays (ELISA) were purchased from R&D Systems (DuoSet, Abingdon, UK). Antibodies were purchased from Calbiochem (heme oxygenase-1) and Biogenesis, Poole, UK (GAPDH). Protease inhibitor cocktail was obtained from Roche Diagnostic (Lewes, UK). All other chemical reagents were obtained from Sigma (Poole, UK).

### Isolation and culture of human airway smooth muscle cells

Human airway smooth muscle was obtained from lobar or main bronchus from patients undergoing lung resection for carcinoma of the bronchus. The smooth muscle was dissected out under sterile conditions and placed in culture as previously described [16]. Cells were maintained in Dulbecco's modified Eagle's medium (DMEM) containing 10% fetal calf serum supplemented with sodium pyruvate (1 mM), L-glutamine (2 mM), non-essential amino acids (1:100), penicillin (100 U/ml)/streptomycin (100 μg/ml) and amphotericin B (1.5 μg/ml) in a humidified atmosphere at 37°C in air/CO_2 _(95:5 % vol/vol). At confluence, HASMC cultures exhibited a typical hill-and-valley appearance. Immunofluorescence techniques for calponin, smooth muscle α-actin and myosin heavy chain revealed that more than 95% of the cells displayed the characteristics of smooth muscle cells in culture. HASMC at passages 3–7 from 9 different donors were used in the studies described below.

### Cigarette Smoke Extract

Cigarette smoke extract (CSE) was prepared by combusting four full strength Marlboro cigarettes (filters removed) through a modified 60 ml syringe apparatus and passing the smoke through 100 mls of DMEM. Each cigarette yielded 5 draws of the syringe (to 60 ml mark), with each individual draw taking approximately 10 seconds to complete. This solution represents '100%' strength. Smoked medium was then passed through a 0.25 μM filter in order to sterilise the solution. Smoked medium was diluted to the required strength in DMEM and placed upon the cells immediately afterwards.

### Cell treatment

Prior to the experiments, confluent cells were growth-arrested by FCS deprivation for 24 h in DMEM supplemented with sodium pyruvate (1 mM), L-glutamine (2 mM), non-essential amino acids (1:100), penicillin (100 U/ml)/ streptomycin (100 μg/ml), amphotericin B (1,5 μg/ml), insulin (1 μM), transferrin (5 μg/ml), ascorbic acid (100 μM) and bovine serum albumin (0,1 %). Cells were then exposed to smoke (0–30%) in the presence and absence of TNFα (1 ng/ml). In additional experiments cells were pretreated with 100 μM glutathione (GSH) for 30 min before exposure to CSE.

### Cell viability

HASMC viability was assessed by the mitochondrial-dependent reduction of 3-(4,5-dimethylthiazol-2-yl)-2,5-diphenyltetrazolium bromide (MTT) to formazan. Cells grown in 96-well plates were treated as indicated above, washed with PBS and 100 μl MTT solution (1 mg/ml) was added to each well. After 1 hour of incubation at 37°C, the MTT solution was removed and the converted dye was solubilized with 100 μl DMSO. The OD was measured using a spectrophotometer set to 550 nm. None of the conditions studied cause visual morphology markers of apoptosis over the time course studied (not shown).

### Cytokine assay

Cell supernatants were harvested 24 hours after stimulation and stored at -70°C until assayed for RANTES, eotaxin and IL-8. Cytokine levels were determined by using specific sandwich enzyme-linked immunosorbent assays (ELISA) according to the manufacturers' instructions.

### RT-PCR and Real-time PCR

Total RNA was isolated from HASMC after 6 hours using the RNeasy Mini Kit (Qiagen, Crawley, UK) according to the manufacturer's instructions. cDNA was generated by reverse transcription (RT) using random hexamers. The cDNA (42 ng/reaction) was used as a template in the subsequent polymerase chain reaction (PCR) analyses. Transcript levels were determined by real-time PCR (Rotor Gene 3000, Corbett Research, Australia) using the Sybre Green PCR Master Mix Reagent Kit (Promega, San Luis Obispo, USA). The sequence for IL-8 PCR primer were sense 5'-GCCAACACAGAAATTATTGTAAAGCTT and antisense 5'-CCTCTGCACCCA GTTTTCCTT'. Primers for GAPDH were sense 5'-ATTCCATGGCACCGT CAAGGCT and antisense 5'-TCAGGTCCACCACTGACACGT. Primers were used at a concentration of 0.5 μM for real-time PCR in each reaction. Cycling conditions for real-time PCR were as follows: step 1, 15 min at 95°C; step 2, 15 sec at 94°C; step3, IL-8: 25 sec at 60°C, GAPDH: 25 sec at 64°C; step 4, 22 sec at 72°C, with repeat from step 2 to step 4 for 40 times. Data from the reaction were collected and analysed by the complementary computer software (Corbett Research, Australia). Relative quantitations of gene expression were calculated using standard curves and normalized to GAPDH.

### Western immunoblot analysis for heme oxygenase-1

Confluent HASMC were exposed to CSE (0–20 %). After 24 hours of incubation, cells were rinsed with ice-cold wash buffer (PBS containing 2 mM PMSF) and scraped off the culture dish. HASMC were pelleted by centrifugation at 1000 RPM at 4°C for 5 min and lysed in radioimmunoprecipitation assay (RIPA) buffer (PBS containing 0.5% sodium deoxycholate, 0.1% sodium dodecyl sulphate (SDS), 1% Igepal and 1 tablet protease inhibitor cocktail 10 ml^-1 ^buffer). Samples were solubilized by sonication followed by centrifugation (10,000 × g, 4°C, 4 min). Protein concentrations were determined using the BCA protein assay kit (Pierce, Rockford, USA). Lysates were boiled for 10 min and total protein extracts (40 μg/lane) were separated by SDS-polyacrylamide gel electrophoresis (SDS-Page) on a 4–12 % acrylamide precast gel (Novex, Invitrogen, Paisley, UK). The separated proteins were transferred electrophoretically to a nitrocellulose membrane in transfer buffer (Novex) and the membrane was then blocked with 5% nonfat dry milk in TBS containing 0.1% Tween 20 (TBST) for at least 1 hour at room temperature. Blots were then incubated overnight at 4°C with an anti-HO-1 antibody in TBST containing 5% dried nonfat milk at a 1:1000 dilution. The next day, the membrane was washed 3 times with TBST and then incubated for 1 hour with a 1:2000 dilution of goat anti-mouse HRP-conjugated secondary antibody in TBST containing 5% nonfat dry milk. The membrane was then washed as before and visualized by enhanced chemiluminescent (ECL) solution (Amersham, Buckinghamshire, UK). Membranes were reprobed with a mouse anti-GAPDH monoclonal antibody (1:5000, Biogenesis, Poole, UK) in order to show the amount of protein loaded. Signals were quantified by scanning densitometry using software from Ultra-Violet Products (UVP) (Cambridge, UK). Densitometry data were normalized for GAPDH values.

### Statistics

Data are presented as mean ± SEM. Data were compared using one-way analysis of variance (ANOVA) followed by Bonferroni's t test post hoc to determine statistical differences. A p value < 0.05 was considered significant. SigmaStat software (Jandel Scientific, Germany) was used for statistical analysis.

## Results

### Effects of cigarette smoke extract on IL-8 expression and protein release

**Figure 1:**
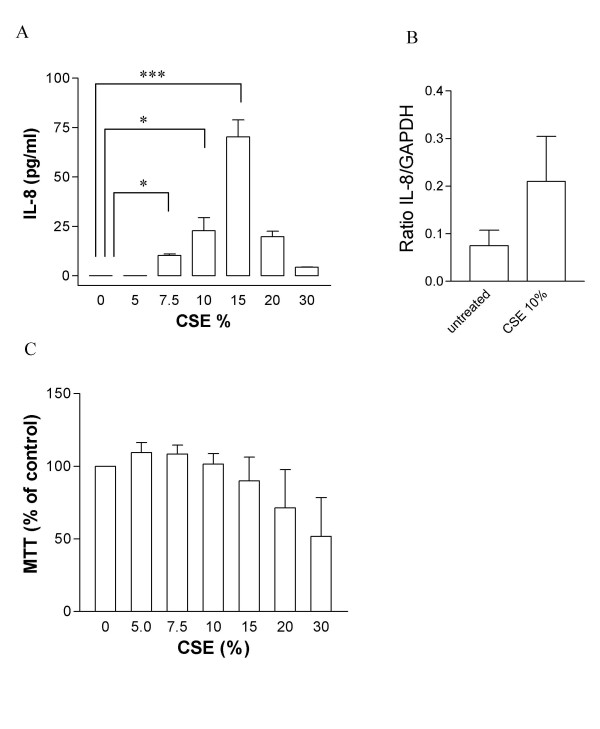
Effect of increasing concentrations of CSE on IL-8 production from HASMC. (A) Cells were stimulated with CSE concentrations from 5–30% for 24 hours. Cell free supernatants were assessed for IL-8 by ELISA. n = 3 from 1 donor. Similar results were obtained from 2 other donors. *** p < 0.001; * p < 0.05 compared to untreated cells. (B) Effect of CSE (10%) on IL-8 mRNA expression in HASMC. Cells from 4 different donors were used for the experiments. Data were normalized to GAPDH expression and are expressed as mean ± SEM. (C) HASMC viability in the presence of CSE (0–30%) was assessed by using the MTT test. Results are expressed as percentage of untreated control cells (mean ± SEM, n = 3).

Under control culture conditions, IL-8 release from HASMC was below the detection limit of the ELISA over the 24-hour experimental period. Increasing concentrations of smoke induced a 'bell-shaped' response curve for IL-8 release by HASMC. Maximum induction of IL-8 release was seen at a concentration of 15 % CSE (baseline 0 pg/ml; 15% CSE 70.3 ± 8.6 pg/ml, p < 0.001; figure 1). However, at concentration of 20% and 30%, the release of IL-8 was lower. In order to assess whether CSE-induced upregulation of IL-8 production from HASMC at up to 15% concentration was the result of increased IL-8 gene transcription, we measured IL-8 mRNA expression by real-time PCR. Stimulation of HASMC with CSE (10%) for 6 hours led to increased IL-8 mRNA expression (Ratio IL-8/GAPDH: baseline 0.075 ± 0.03, 10% CSE 0.21 ± 0.09, figure 1). Viability of cells exposed to CSE remained unchanged up to concentrations of 15 % (104.8 ± 3.2 % of control) but declined at concentrations of 30% cigarette smoke (50.1 ± 9.7 % of control, figure 1).

### Role of oxidative stress in cigarette smoke-induced IL-8 release

**Figure 2:**
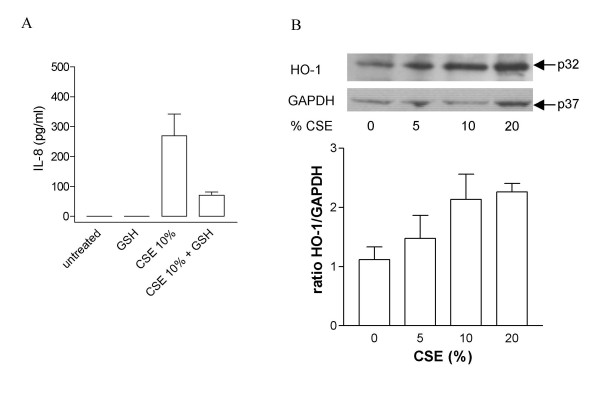
(A) Effect of glutathione (GSH) on cigarette smoke-induced IL-8 release from HASMC. Cells were pretreated with 100 μM GSH for 30 min before adding CSE (10%). Data are expressed as mean ± standard error of the mean (SEM). (B) CSE induced heme oxygenase-1 (HO-1) expression in HASMC. Cells were exposed to CSE (0–20%) for 24 hours. HO-1 expression was detected by western blotting. The blot shown in the upper panel was stripped and reprobed using a GAPDH antibody to show equal protein loading. A representative example of three identical experiments is shown. In the lower panel densitometric analysis of HO-1 expression, normalized by GAPDH expression, is shown.

The stimulatory effects of CSE were greatly inhibited by pre-treatment of cells with GSH (100 μM; 10 % CSE 270.8 ± 72.5 pg/ml, 10 % CSE + GSH 70.9 ± 10.8 pg/ml, figure 2), which quenches extracellular oxidative stress [17]. Heme oxygenase-1 is expressed in most cell types and is highly inducible by oxidative stress. In order to investigate whether CSE exposure causes an intracellular oxidative stress response in HASMC, we measured the expression of heme-oxygenase-1 levels before and after exposure to smoke by western blot analysis. HASMC expressed detectable levels of heme-oxygenase-1 when cultured under control conditions. However, heme-oxygenase-1 levels were increased when cells were treated with CSE (5–20 %; figure 2).

### Effect of cigarette smoke extract and TNFα on IL-8 release

**Figure 3:**
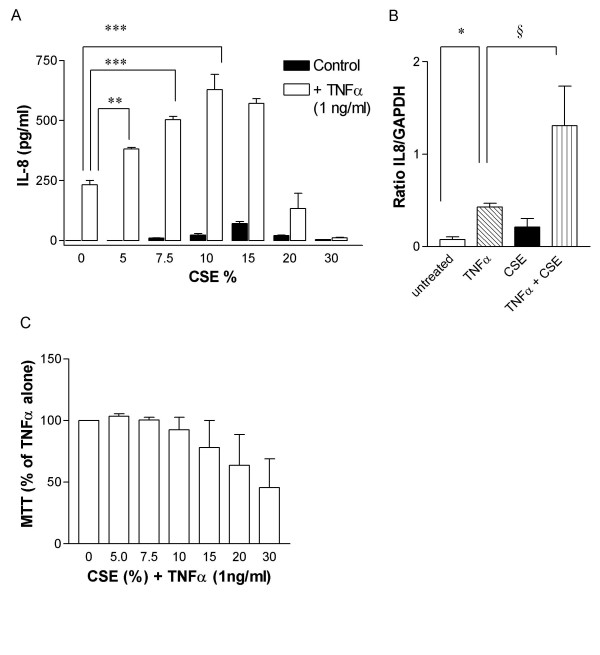
CSE synergises with TNFα (1 ng/ml) in inducing IL-8 release and expression in HASMC. (A) Cells were stimulated with CSE concentrations from 5–30% for 24 hours in the absence and presence of TNFα (1 ng/ml). IL-8 in the cell-free supernatant was measured by ELISA. The results shown are those from 3 replicate measurements from cells obtained from one donor. Similar results were obtained from 2 other donors. *** p < 0.001; ** p < 0.01 compared to cells treated with TNFα only. (B) IL-8 mRNA expression in HASMC exposed to CSE (10%) in the presence and absence of TNFα (1 ng/ml). Cells from 4 different donors were used for the experiments. Data were normalized to GAPDH expression and are expressed as mean ± SEM. * p < 0.05 compared to untreated cells; § p < 0.05 compared to cells treated with TNFα only. (C) HASMC viability in the presence of TNFα (1 ng/ml) alone or in combination with CSE (0–30%) was assessed by using the MTT test. Results are expressed as percentage of cells treated with TNFα only (mean ± SEM, n = 3).

TNFα induced a concentration dependent release of IL-8 from HASMC at 0.1 to 10 ng/ml (not shown), with 1 ng/ml representing an approximate EC_50 _concentration. Furthermore, TNFα (1 ng/ml) acted in synergy with CSE on the release of IL-8 from HASMC (figure 3). This synergy was observed across the concentration range of 5–15% CSE with maximum effect seen at 10% smoke (TNFα 232.1 ± 18.4 pg/ml, 10% CSE + TNFα 628.5 ± 64.2 pg/ml, p < 0.001, figure 3). This synergy was lost with CSE 20% and in fact at 30% there was inhibition of IL-8 release. In line with protein release, TNFα synergised with CSE (10%) in induction of IL-8 mRNA (figure 3). Cell viability remained unchanged in cells treated with either TNFα alone or in combination with CSE at concentrations of up to 10%. AT CSE 15%, cell viability declined mildly with further reduction seen at CSE 20–30% (Figure 3).

### Effect of cigarette smoke extract on eotaxin and RANTES release

**Figure 4:**
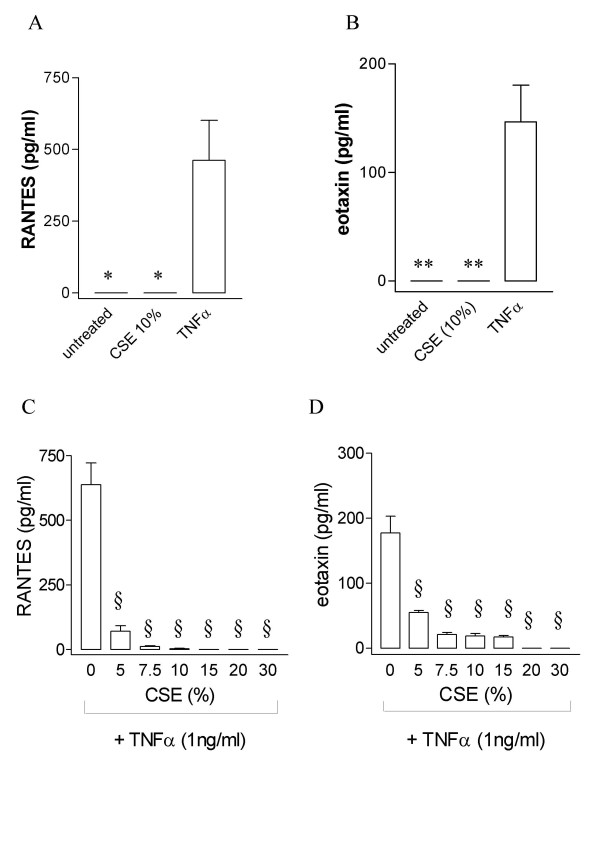
Effect of CSE or TNFα on (A) RANTES and (B) eotaxin release from HASMC. Cells were incubated in the presence and absence of CSE (10 %) or TNFα (1 ng/ml) for 24 hours. Effect of CSE on TNFα-induced (C) RANTES and (D) eotaxin release from HASMC. Cells were stimulated with CSE concentrations from 5–30% for 24 hours in presence of TNFα (1 ng/ml). Cell free supernatant was assessed for RANTES and eotaxin by ELISA. Cells from 3 different donors were used for the experiments. Data are expressed as mean ± SEM.

Similar to observations made with IL-8 release, levels of either eotaxin or RANTES were below the level of detection in medium from cells cultured under basal conditions. Similar to IL-8, incubation of cells with TNFα (1 ng/ml) for 24 hours induced increased levels of both eotaxin and RANTES released by the cells (Figure 4; RANTES 637.5 ± 84.5 pg/ml; eotaxin 177.1 ± 25.9 pg/ml). However, by contrast to IL-8, CSE at all concentrations (5%–30%) failed to induce release of either eotaxin or RANTES from HASMC (figure 4). Furthermore, CSE did not synergise with TNFα in the release of either eotaxin or RANTES. In fact, CSE inhibited the release of these chemokines when induced by TNFα (figure 4) This effect was not reversed by pre-treatment of HASMC with GSH (100 μM) before adding cigarette smoke solution (data not shown).

## Discussion

Cigarette smoke extracts up to 20% activated HASMC to release IL-8, an important mediator of neutrophilic inflammation in COPD, and enhanced the release of IL-8 induced by TNFα. The effect of CSE on IL-8 production was associated with enhanced IL-8 gene transcription and increased expression of HO-1, an indicator of intracellular oxidative stress. By contrast, CSE had no effect on release of the eosinophil chemotactic chemokines eotaxin and RANTES at baseline levels and potently inhibited TNFα-induced release of these chemokines. These results indicate that HASMC may release IL-8 directly on contact with cigarette smoke extracts and contribute to airway neutrophilic inflammation in COPD; the overexpression of TNFα in COPD may augment this response. In addition, the effect of cigarette smoke on HASMC may also explain why there is little eosinophilic response in COPD since the release of RANTES and eotaxin which have eosinophilic chemotactic effects is inhibited. Our hypothesis of selective induction of neutrophil accumulation in the lungs by smoke is supported by a recent study where [18] cigarette smoke increased neutrophil but reduced eosinophil numbers in the lavage fluid of ovalbumin-sensitized mice.

Neutrophils are considered as important inflammatory cells in the pathogenesis of COPD because of their ability to release various substances with harmful effects on lung structures, such as oxidants, cytokines and especially proteases [19,20]. Various mechanisms may account for the accumulation of neutrophils in the lungs of cigarette smokers such as delayed transit time, reduced apoptosis or enhanced migration from the vasculature due to increased expression of adhesion molecules [21-24]. In addition, cigarette smoke induces the release of IL-8, a potent chemotactic factor and activator for neutrophils [25]. Elevated levels of IL-8 were found in the BAL-fluid of smokers compared to nonsmokers and correlated positively with neutrophil counts in BAL fluid of smokers [8]. Cigarette smoke induces IL-8 production from various pulmonary residential cells such as monocytes, macrophages, epithelial cells and fibroblasts [6-8,26]. In line with this notion, we show here that cigarette smoke is a potent stimulus for IL-8 production from HASMC. The increased levels of IL-8 release were directly associated with increases in IL-8 gene expression, as measured by real time PRC. It should be noted, however, that the effects on mRNA levels could be due to either increased gene expression or increased message stability. The in-vivo relevance of this observation is demonstrated by a recent study which showed infiltration of the airway smooth muscle layer with neutrophils in patients with COPD [27].

While the release of IL-8 induced by cigarette smoke extract up to 20% alone was modest, this effect was potentiated by TNFα. Smoke alone, or smoke together with TNFα, also induced gene transcription of IL-8. The ability of cigarette smoke to release IL-8 in the presence of TNFα may explain the persistence of neutrophilic inflammation in cigarette smokers, where inflammatory cytokines, including TNFα, are usually present in the lung [28]. In mice, TNFα appears to be a central mediator for smoke-induced inflammation and connective tissue breakdown [29].

By contrast, CSE did not induce the release of eotaxin or RANTES. In fact, it inhibited the release of these chemokines in TNFα-stimulated cells, an effect that could not be explained by reduced cell viability and was not reversed by addition of the antioxidant GSH. Interestingly, we also observed smaller levels of IL-8 release with higher concentrations of CSE above 20%. This was not entirely due to cell death. In addition the potentiation of TNFα release of IL-8 was lost, and indeed at 30% concentration there was almost complete inhibition of IL-8 release. Because cigarette smoke is a complex insult consisting of more than 4000 different components [30], it is likely that at high concentrations of CSE, some components achieve concentrations that have inhibitory effects on IL-8 release overriding the stimulatory effects of other components of cigarette smoke. Inhibition of eotaxin and RANTES release at CSE concentrations which stimulated HASMC to release IL-8 indicates that there is differential sensitivity to the effects of smoke among the various subsets of chemokines. High concentrations of CSE are less likely to be relevant in vivo. Although it is not known what concentration of CSE airway smooth muscle cells are exposed to *in vivo*, it is likely to be a diluted concentration.

Although the mechanisms involved in the development of smoke-induced lung diseases are not fully understood, it is widely accepted that oxidative stress is a key factor responsible for lung destruction seen in smokers. For example, oxidants inactivate α_1_-antitrypsin, the major protease inhibitor in the lung [31] and reactive oxygen species induce infiltration of neutrophils into the lung [32], which are an important source of oxidants themselves. Our results support the theory that oxidative stress plays an important role in smoke-related lung diseases. HASMC exposed to cigarette smoke showed increased expression of heme-oxygenase-1, an intracellular indicator of oxidative stress. In addition, GSH, an important intra- and extracellular antioxidant in the lung, inhibited cigarette smoke-induced IL-8 release in HASMC. Interestingly, the inhibitory effect of cigarette smoke on inhibition of RANTES and eotaxin was not dependent on oxidative stress. The mechanisms involved in CSE mediated inhibition of chemokine production remain to be identified. However, our observations are in line with others showing that the release of eotaxin [33,34] and IL-8 can be differencial regulated by inflammatory or anti-inflamamtory stimuli [35].

## Conclusion

It is important to understand how smoking mediates airway inflammation in order to identify possible targets for treatment of patients with chronic obstructive pulmonary disease. The present study demonstrates that cigarette smoke stimulates the release of the neutrophil chemotactic cytokine IL-8 but inhibits the production of the eosinophil chemotactic factors eotaxin and RANTES in HASMC. Considering the anatomical location of the airway smooth muscle with proximity to the vasculature, the data from our study suggest that HASMC play an important role in promoting neutrophil migration from the vasculature to the interstitium in lung diseases associated with cigarette smoke and may help to explain why COPD unlike asthma, is predominantly associated with neutrophil recruitment.

## Competing interests

The author(s) declare that they have no competing interests.

## Authors' contributions

UO prepared primary cultures of HASMC, carried out cytokine assays, RT-PCR and real-time PCR, western blotting for heme-oxygenase 1, performed statistical analysis and drafted the manuscript.

FC participated in the design, coordination of the study and drafting of the manuscript

MW prepared cigarette smoke extract, participated in cell treatment and carried out cell viability assays.

MJ participated in drafting the manuscript.

JAM is the chief investigator who conceived the study.

All authors read and approved the final manuscript.
